# Molecular Therapeutics for Diabetic Kidney Disease: An Update

**DOI:** 10.3390/ijms251810051

**Published:** 2024-09-19

**Authors:** Man Guo, Fangfang He, Chun Zhang

**Affiliations:** Department of Nephrology, Union Hospital, Tongji Medical College, Huazhong University of Science and Technology, Wuhan 430022, China

**Keywords:** diabetic kidney disease, therapeutics, miRNA, stem cell, gene therapy, gut microbiota

## Abstract

Diabetic kidney disease (DKD) is a common microvascular complication of diabetes mellitus (DM). With the increasing prevalence of DM worldwide, the incidence of DKD remains high. If DKD is not well controlled, it can develop into chronic kidney disease or end-stage renal disease (ESRD), which places considerable economic pressure on society. Traditional therapies, including glycemic control, blood pressure control, blood lipid control, the use of renin–angiotensin system blockers and novel drugs, such as sodium–glucose cotransporter 2 inhibitors, mineralocorticoid receptor inhibitors and glucagon-like peptide-1 receptor agonists, have been used in DKD patients. Although the above treatment strategies can delay the progression of DKD, most DKD patients still ultimately progress to ESRD. Therefore, new and multimodal treatment methods need to be explored. In recent years, researchers have continuously developed new treatment methods and targets to delay the progression of DKD, including miRNA therapy, stem cell therapy, gene therapy, gut microbiota-targeted therapy and lifestyle intervention. These new molecular therapy methods constitute opportunities to better understand and treat DKD. In this review, we summarize the progress of molecular therapeutics for DKD, leading to new treatment strategies.

## 1. Introduction

Diabetic kidney disease (DKD) is a chronic disease caused by diabetes mellitus (DM). The early manifestation of DKD is microalbuminuria, which gradually progresses to massive proteinuria and progressive renal dysfunction. If DKD is not well controlled, it eventually progresses to end-stage renal disease (ESRD) [1], which is the main cause of increased morbidity and mortality in diabetic patients. Because DKD is a common complication of DM, and the prevalence of DM is increasing worldwide, the prevalence of DKD has also increased. Approximately 40% of patients with type 2 DM (T2DM) and 30% of patients with type 1 DM (T1DM) progress to DKD [2,3,4]. More than 700 million people worldwide are estimated to be currently affected by DKD, and this number will continue to increase in the coming decades [5]. The high medical costs and poor prognosis of people in end-stage DKD have placed a heavy burden on society and families [6,7].

The pathogenesis of DKD is complex, and the therapeutic targets are not yet clear. Traditionally, glomerular hypertension, altered renal hemodynamics, inflammation, oxidative stress stimulation, and activation of the renin–aldosterone system are believed to contribute to the progression and poor prognosis of DKD [8]. At present, the treatment methods for DKD mainly include classic methods, such as glycemic control, blood lipid control, blood pressure control, and other symptomatic supportive treatment methods. In addition, various drugs, such as metformin, mineralocorticoid receptor antagonists (MRAs), sodium–glucose cotransporter 2 (SGLT2) inhibitors, and glucagon-like peptide-1 receptor (GLP-1R) agonists, have also entered clinical use. However, the benefits of these treatments for DKD remain limited and they cannot prevent DKD from progressing into to ESRD. We need to search for new targets and strategies for treating DKD.

In recent years, treatment methods for DKD have also been continuously advancing. As research on new treatment methods, such as gene therapy, stem cell therapy, miRNA therapy, gut microbiota therapy and lifestyle therapy has progressed, new options have been provided for the treatment of DKD. This study focuses on the therapeutic potential of the above therapies for DKD to provide a new theoretical basis for the clinical treatment of DKD.

## 2. Conventional Treatment and Newer Medications for DKD

In the past 20 years, renin–angiotensin–aldosterone system (RAAS) blockers, angiotensin-converting enzyme inhibitors (ACEIs) and angiotensin receptor blockers (ARBs) have been the only drug choices for the treatment of DKD [9]. The above treatment has limited efficacy in preventing the progression of DKD. With the development of therapeutic targets for DKD, new therapeutic drugs for DKD are being clinically applied.

### 2.1. Renin–Angiotensin–Aldosterone System Blockades

RAAS inhibitors are the oldest and most advanced drugs used for the treatment of DKD. In many clinical trials, RAAS inhibitors have been proven to effectively treat DKD. ACEIs and ARBs can reduce glomerular hypertension, prevent structural damage to glomeruli, and reduce proteinuria levels by blocking the formation and function of angiotensin II [10]. These drugs have been shown to have renal and cardiovascular protective functions [11] and can prevent the progression of renal dysfunction in patients with diabetic or nondiabetic kidney disease [12].

### 2.2. Mineralocorticoid Receptor Antagonists

The activation of mineralocorticoid receptors can lead to renal inflammation and fibrosis reactions [13], whereas MRAs inhibit aldosterone binding and its subsequent biological effects by inducing different conformational changes in the mineralocorticoid receptor [14]. Clinical trial results suggested that finerenone, a novel nonsteroidal MRA, reduced the risk of renal damage and cardiovascular events in patients with DKD. Guidelines recommend the use of nonsteroidal MRAs as an adjunct therapy in patients with normal potassium levels <4.8 mmol/L, who are already taking SGLT2 inhibitors and RAAS blockers, as well as in patients at high risk of DKD progression with proteinuria (>30 mcg/mg) [13,15].

### 2.3. Glucagon-like Peptide-1 Receptor Agonist

Glucagon-like peptide-1 (GLP-1) is an intestinal insulinotropic hormone secreted by lower intestinal L cells that regulates glycemic levels by activating GLP-1R in the pancreas [4]. Research has shown that GLP-1R agonists exert renal protection in T2DM patients by reducing proteinuria and improving the impaired estimated glomerular filtration rate (eGFR) [16]. Recent experiments have further demonstrated the ability of GLP-1R agonists to reduce the occurrence of cardiovascular complications in DKD patients, making this drug a potential early option for use in DKD patients [9]. In addition, these drugs have the additional benefit of inducing weight loss [9], and KDIGO recommends adding GLP-1R agonists for patients who are already using metformin and SGLT2 inhibitors, but have not achieved their glycemic control goals [17].

### 2.4. Sodium–Glucose Cotransporter 2 Inhibitors

SGLT2 inhibitors are novel oral hypoglycemic drugs that activate the tubuloglomerular feedback mechanism to reduce glomerular hyperfiltration, especially when used in combination with ACEIs or ARBs [18]. SGLT2 inhibitors can inhibit the reabsorption of glucose in the proximal tubules and promote the excretion of urinary glucose [19]. In addition, SGLT2 inhibitors reportedly reduce the production of circulating inflammatory factors, such as interleukins (IL) -6, TNF receptor-1, and TNF receptor-2, and decrease the production of ketones [20]. Many clinical trials have shown that SGLT2 inhibitors can reduce the risk of DKD progression and renal failure, as well as heart failure, atherosclerotic cardiovascular disease and death [21,22,23,24,25]. SGLT2 inhibitors are now recommended as first-line treatments for DKD patients.

## 3. Gene Therapy

The pathological process of DKD is complex, and the core mechanism involves inflammation and oxidative stress induced by high glucose in the blood, resulting in damage to tubules and glomeruli which ultimately leads to kidney injury and fibrosis [8]. Therefore, the treatment of inflammation and oxidative stress is very valuable in DKD, which may lead to a potential new strategy for DKD treatment.

### 3.1. Inflammation-Based Gene Therapy

Increasing evidence shows that inflammation is a key factor in the pathogenesis of DKD [26,27]. In recent years, regulating the inflammatory response has become a new method to prevent the development of DKD. At present, therapeutic targets based on inflammatory molecules, including chemokines, adhesion factors, cytokines, and intracellular signaling pathways involved in inflammatory responses have been surveyed [28].

#### 3.1.1. Adhesion and Chemokine Molecules Inhibition

Adhesive molecules are proteins on the surface of cells that participate in binding cells together or attaching them to the extracellular matrix [27]. Research has shown that various adhesion molecules, such as ICAM1, VCAM-1, and VAP-1, are involved in the occurrence and development of DKD, mainly by increasing the excretion of urinary proteins [29,30,31]. At present, treatments for adhesion factors remain limited. A Phase II clinical trial revealed that the oral VAP-1 inhibitor ASP8232 could delay the progression of kidney injury [32].

Chemokines and their receptors are soluble small-molecule proteins that play crucial roles in mediating the migration of immune cells to renal tissue and subsequently triggering inflammatory responses [33]. In DKD, the chemokine C-C motif-ligand 2 (CCL2), drives the recruitment of T cells and macrophages to the kidneys, and promotes the occurrence and development of DKD by binding to chemokine receptor 2 (CCR2) [34]. A Phase II clinical trial revealed that emapticap pegol could improve proteinuria and exert renal protection by blocking the CCL2-CCR2 signaling pathway [35]. In addition, CXCL12, a chemokine produced by podocytes, has also been reported to cause proteinuria and glomerulosclerosis in diabetic mice. NOX-A12, a CXCL12-specific inhibitor, can improve proteinuria and glomerulosclerosis in db/db mice [36]. However, relevant clinical research needs to be carried out.

#### 3.1.2. Inhibiting Inflammatory Cytokines

Cytokines, including interleukins and interferons, are immune modulators that play a vital role in the occurrence of DM-related complications. Currently, many cytokines are involved in DKD research, including IL-1β, IL-18, and TNF-α [37], which trigger an inflammatory response in DKD. Specific compounds that target these inflammatory factors have been reported. Research has shown that alprostadil and thrombomodulin lectin domains alleviate inflammation in DKD by inhibiting the expression of IL-18 [38,39]. The inhibition of TNF-α with infliximab could reduce the excretion of urinary albumin in a streptozotocin (STZ)-induced diabetic rat model [40]. Neutralizing IL-1β antibodies improved the level of blood glucose and reduced inflammation in T2DM patients by restoring insulin production [41]. However, a clinical trial suggested that canakinumab, an inhibitor of IL-1β, did not provide significant clinical benefits or cause substantial harm in patients with chronic kidney disease (CKD) [42]. Anti-inflammatory therapy targeting specific molecules may be a promising treatment strategy for DKD.

#### 3.1.3. JAK/STAT Inhibitors

Numerous studies have shown that the Janus kinase (JAK)/signal transducer and activator of transcription (STAT) signaling pathway is associated with the progression of DKD. JAK/STAT mediates the occurrence and development of DKD by inducing the transcription of genes that encode inflammatory factors, adhesion molecules, growth factors, extracellular matrix proteins, and pro-oxidant enzymes [37]. At present, many selective compounds, such as AG-490 which targets JAK2, fludarabine which targets STAT1, and nifuroxazide which selectively acts on STAT3, have been reported to alleviate kidney damage by inhibiting hyperglycemia-induced damage to the kidney [43,44,45]. A Phase II clinical trial revealed that balitinib, a selective inhibitor of JAK1 and JAK2, reduced proteinuria in DKD patients [46]. These findings suggest that targeting JAK/STAT has broad prospects in the treatment of DKD.

#### 3.1.4. Inhibition of NF-κB

NF-κB is a key transcription factor in the pathogenesis of the inflammatory response in DKD, and activation of NF-κB can induce the expression of various inflammatory mediators, which play an important role in the pathogenesis of DKD [47]. Celastrol, an inhibitor of NF-κB, can improve insulin resistance and play an anti-inflammatory role, making it a new useful method for treating DKD [48]. In addition, the NF-κB inhibitor, BAY 11-7082 and a small molecule analog of resveratrol, have been reported to reduce renal inflammation in mouse models of DKD by inhibiting the activation of NF-κB, exerting renoprotective effects. However, owing to the complexity of NF-κB signaling [49,50], a clinical study revealed that PTX indirectly inhibited the activity of downstream NF-κB signaling by suppressing phosphodiesterase activity and activating protein kinase A/cAMP response element binding protein, reducing the progression of proteinuria in DKD [26,51]. At present, specific drugs that target NF-κB still need further research.

### 3.2. Antioxidative Gene Therapy

The oxidative stress process during DKD is caused by increased reactive oxygen species (ROS) induced by peroxidase in a high-glucose environment, accompanied by the depletion of antioxidants, which results in an imbalance of the oxidative/antioxidant system and causes cell and tissue damage [52]. The regulation of antioxidant genes is highly important for controlling the occurrence and development of DKD.

#### 3.2.1. NOX Inhibitors

Nicotinamide adenine dinucleotide phosphate (NADPH) oxidase (NOX) is the most important source of ROS in diabetic patients [53]. Inhibiting NOX expression can reduce the production of ROS and is considered a new option for the treatment of DKD. Animal experiments revealed that the deletion of the NOX4 gene in DKD mice significantly reduced the production of ROS in the renal cortex and protected the kidney from damage [54]. GKT137831 is a small molecule inhibitor of NOX1 and NOX4. GKT137831 provided a similar degree of renal protection as NOX4 knockout in diabetic mice [55]. However, in a clinical trial, it failed to effectively reduce proteinuria (NCT02010242). Recently, a new pan-nitrogen oxide inhibitor, APX-115, was shown to reduce proteinuria, glomerular hypertrophy, renal tubular injury, podocyte injury, fibrosis, inflammation and oxidative stress to prevent renal injury in diabetic mice [56]. However, further clinical trials are needed to evaluate the clinical benefits of APX-115 for DKD patients.

#### 3.2.2. AGE Inhibitors

Under high-glucose conditions, the body produces a large amount of advanced glycation end products (AGEs), which subsequently bind to AGE receptors (RAGE) to active downstream target molecules, such as NOX, and produce a large amount of ROS [52]. AGE inhibitors, such as aminoguanidine, pyridoxamine, and alagebrium, have been shown to protect the kidneys. However, these therapies have not yet been translated into clinical practice. Another method for targeting the AGE/RAGE axis is focused on inhibiting the biological activity of AGEs by interfering with RAGE. RAGE inhibitors, such as azeliragon, have been developed for the treatment of Alzheimer’s disease [57]. In 2018, a Phase II randomized controlled trial was launched on the effects of green tea extract on soluble RAGE and kidney disease in patients with T2DM (NCT03622762), but the status of this study remains unclear.

#### 3.2.3. Activation of Nrf2 Pathways

Nrf2 is a key regulatory factor in the cellular antioxidant response. When cells are stimulated by ROS, Nrf2 enters the nucleus and can activate the expression of various downstream target proteins, such as heme oxygenase-1 and superoxide dismutase. These activated target proteins can regulate the redox balance in the body, to restore the body from an oxidative state to a normal level [58]. In vitro experiments have shown that Nrf2 knockout induces ROS production by upregulating the expression of NOX2 [59]. In diabetic mouse models, dietary supplementation with Nrf2-targeted compounds, such as sulforaphane or cinnamic aldehyde, can alleviate the progression of DKD by reducing the oxidative stress response [60]. Bardoxolone methyl is an activator of Nrf2, whereas a Phase 3 study (AYAME study) in patients with DKD was terminated because the early instances of heart failure in patients participating in the study were surprisingly increased [61]. Further in-depth studies of novel Nrf2 regulators are highly valuable.

### 3.3. Antifibrotic Therapy

The inflammatory response and oxidative stress are also involved in activating the excessive production and deposition of extracellular matrix proteins in renal tissue, leading to renal fibrosis, which is the main outcome of almost all CKD cases and causes irreversible kidney damage. The TGF-β1 signaling pathway is the core molecule of renal fibrosis in DKD and can regulate fibrosis by promoting the overexpression of the extracellular matrix, which enhances the dedifferentiation of tubular epithelial cells and glomerular endothelial cells, and promotes cross-linking between collagen and elastin fibers [4,62]. The renoprotective effects of conventional therapies for DKD, such as RAAS inhibitors (including ACEIs, ARBs, and MRAs) are, at least partially, attributed to a reduction in TGF-β signaling. In animal experiments, TGF-β neutralizing monoclonal antibodies and drugs such as pirfenidone effectively alleviated renal fibrosis by reducing the activity of the TGF-β1 gene promoter and the release of the TGF-β1 protein in DKD [63,64,65]. Due to the complexity of TGF-β molecules, targeting them may inhibit important physiological functions, such as systemic anti-inflammatory activity. Therefore, current efforts have shifted toward targeting the intrinsic downstream components and molecules that can affect the profibrotic activity of the TGF-β pathway, such as SMAD proteins and related miRNAs [66]. A new strategy for reducing pathologically high levels of TGF-β activity to physiological levels has potential value for developing more effective and safe treatment methods to specifically delay the progression of DKD [66].

Overall, gene therapy can modify specific genes, which is expected to completely cure the disease, and an increasing number of clinical trials that target related genes are being conducted (Table 1). However, gene therapy still has limitations, theoretical research on gene therapy is still relatively weak, and gene technology is difficult, unstable, and has significant differences in effectiveness. Developing other novel treatment strategies to provide more possibilities for the prevention and treatment of DKD is necessary.

## 4. MiRNA Therapy

MiRNAs are single-stranded small-molecule RNAs that are highly conserved, tissue specific and tissue targeted [67]. MiRNAs in the human genome regulate more than 60% of human protein-coding genes [68] and regulate gene expression at the post-transcriptional level by forming RNA-induced silencing complexes that recognize the 3′ UTRs of target mRNAs. They degrade target mRNAs when fully paired and inhibit translation when incompletely paired, resulting in negative regulation of target genes [69]. MiRNAs have been shown to regulate various biological processes and are closely related to the occurrence and development of DKD [70]. Currently, various RNA family members, such as the miR-192, miR-21, and miR-29 families, are reportedly specifically overexpressed in DKD renal tissue and play key roles in regulating glomerular basement membrane injury, mesangial damage, podocyte damage, and renal interstitial fibrosis [71]. Although clinical studies targeting miRNAs in DKD are limited, laboratory findings provide new possibilities for small-molecule therapy in DKD. Thus, miRNA inhibitors, agonists, and mimics that control the expression levels of miRNAs can be developed for use in DKD patients in the future [72].

### 4.1. MiRNA Inducers

MiRNA inducers are small molecules that can upregulate the expression of target miRNAs. At present, many studies have focused on the role of miRNA inducers in protecting renal function in DKD. Triptolide is a monomeric compound, that is isolated from traditional Chinese medicine. Han et al. reported that triptolide could induce the expression of miRNA-137, reduce the level of proteinuria in DKD rats by inhibiting the activation of the Notch1 pathway, and improve glomerulosclerosis [73]. Linagliptin, a dipeptidyl peptidase-4 inhibitor, can delay the progression of renal fibrosis by increasing the level of miR-29a [74]. C66, a new analog of curcumin, has been reported to increase the expression of miR-200a, upregulate the level of Nrf2, reduce renal oxidative damage caused by DM, and play an important role in renal protection [75]. Currently, more clinical trials are needed to validate the efficacy of drugs based on miRNA induction, and drugs that induce the high expression of miRNAs with protective effects will provide us with another option for treating DKD.

### 4.2. MiRNA Mimics

MiRNA mimics are double-stranded RNA oligonucleotides that mimic endogenous miRNAs. They play a vital role in enhancing or rebuilding endogenous miRNAs [76]. MiRNA mimics with nucleotide sequences are similar to mature miRNAs and can be effectively delivered to cells through lipid reagents or electroporation and then bind to specific genes and to regulate the expression of miRNAs [77,78]. Many studies have focused on the potential application prospects of miRNA mimics in DKD. Cheng et al. reported that supplementation with miR-122-5p mimics could alleviate renal tubular injury and interstitial fibrosis in STZ-induced DKD model mice, reduce urinary creatinine levels, and exert renoprotective effects [79]. Bai et al. reported that the transfection of miRNA-20 mimics could inhibit high glucose-induced inflammation and apoptosis in renal tubular cells, alleviate high glucose-induced damage to renal tubular cells, and serve as a potential therapeutic target for DKD [80]. Clinical trials related to miRNA mimics are already underway, with MRG-201 (an LNA miRNA-29b mimic) inhibiting collagen expression and fibrosis development in skin incision wounds by restoring the levels of targeted miRNAs [81]. In addition, MRX34 (a miRNA-34a mimic) has also entered clinical trials for hepatocellular carcinoma, renal cell carcinoma, and melanoma [82]. Therefore, regulating or administering protective miRNA mimics could become a new approach for the treatment of DKD, with good research value and feasibility.

### 4.3. Small-Molecule Inhibitors

Some small molecules that inhibit or downregulate the expression of target miRNAs are also used in research on DKD. Paclitaxel (used for cancer chemotherapy), a mitotic inhibitor, has been shown to downregulate the expression of miR-192, thereby improving fibrosis in the residual kidney [83]. Hyperoside is the active component of Betula platyphylla flowers and can improve glomerulosclerosis in DKD by downregulating miR-21 to increase the expression of MMP-9 [84]. Wang et al. reported that Astragaloside IV (a bioactive saponin extracted from Astragalus root) could inhibit the expression of miR-21 and miR-192 in DKD models and alleviate renal fibrosis by acting on the TGF-β1/Smad pathway [85,86]. Jia et al. reported that icariin (dried stem and leaf extracts of epimedium) promoted the expression of GLP-1R by downregulating miR-192-5p and exerting renal protection by regulating glucose metabolism [87]. The above research suggests that high-throughput screening of drugs that can reduce the expression of miRNAs may be another option.

### 4.4. MiRNA Sponges

MiRNA sponges are RNA structures that contain multiple miRNA binding sites, that bind miRNAs and prevent them from performing their functions. Source mRNA targets are used to manipulate miRNAs at the cellular level [88]. By subcloning the miRNA binding site into a vector containing 50 and 30 stem loop elements of the U6 snRNA promoter, the sponge can be introduced into cells [89]. Research has shown that the expression of the miR-21 sponge inhibits endogenous miR-21, prevents high glucose-induced PTEN downregulation and Akt phosphorylation, and reduces the expression of fibronectin in the kidney [90].Moreover, circRNAs, which are covalently closed RNA loops produced by reverse-splicing pre-mRNAs, can also act as miRNA sponges [91]. Peng et al. reported that circRNA_010383, a natural sponge of miRNA-135a, inhibited the function of miR-135a by directly binding to miR-135a, leading to proteinuria and renal fibrosis in DKD [92]. Another type of circRNA, circ-AKT3, can act as a sponge of miR-296-3p to reduce the proliferation and fibrosis of mesangial cells in DKD [93], which provides a potential basis for the construction of additional miRNA sponges. At present, an increasing number of new strategies are being used to modify and reverse pathological changes in miRNA expression. In-depth research on miRNA sponges will undoubtedly promote the development of new therapeutic methods.

### 4.5. Anti-miRNA Oligonucleotides

Antisense oligonucleotide (ASO), also referred to as an antagomir, is a method that mainly blocks miRNA binding to target sequences by providing perfectly matched antisense nucleotide chains. Chemical modifications, such as 2′-O-methylation, 2′-O-methoxyethyl, 2′ fluoro-chemistries and locked nucleic acids (LNAs), can be used to improve the affinity of ASOs for target miRNAs and resistance to nuclease degradation in an effort to stably and effectively deliver them into cells [71,94,95]. Among these modifications, LNAs have the best affinity for binding to complementary RNA [96]. At present, relevant animal experiments have revealed the enormous potential of anti-miRNA oligonucleotides in the treatment of DKD. The injection of LNA-modified anti-miR-192 into diabetic mice, effectively inhibited the expression of miR-192 and downstream miRNAs (miR-216a, miR-217 and miR-200 families) and reduced the expression of collagen, TGF-β, fibronectin and p53, thereby alleviating renal fibrosis and proteinuria in DKD [72,97,98]. Antagomirs of miRNA-27a and miRNA-132 have also been reported to reduce collagen deposition, inhibit the progression of renal fibrosis, and alleviate the progression of DKD [99,100]. In addition, knocking down miR-29c with a specific 2′-O-methylation-modified ASO significantly reduced proteinuria and the accumulation of the renal mesangial matrix in DKD model mice, thereby reducing the incidence of DKD [101]. Notably, relevant clinical drugs have also entered the clinical trial stage. Miravirsen (LNA and phosphorothioate-modified antagomir targeting miR-122) and RG-012 (2′-o-methoxyethoxy-modified antagonist targeting miR-21) have shown efficacy in treating hepatitis C [102,103]. The targeted and specific regulation of miRNAs through anti-miRNA oligonucleotides may be a new approach for treating DKD.

The development of effective delivery methods and mechanisms targeting specific renal cell populations is an important challenge for successfully translating miRNAs into clinical applications. In addition, the combination of miRNAs with existing treatment methods such as ACEi and ARBs has provided new therapeutic potential, and further research is necessary to determine whether these combinations can exert synergistic effects [104].

## 5. Stem Cell Therapy

Stem cells are primitive undifferentiated cells with self-replication and multipotent differentiation potential [105]. Stem cells can be classified into embryonic stem cells, adult stem cells, and induced pluripotent stem cells based on their origin [106]. Adult stem cells can be further divided into endothelial progenitor cells, mesenchymal stem cells (MSCs), and osteogenic stem cells. Research has shown that stem cells can protect the kidneys through direct differentiation, paracrine effects, and other mechanisms. Stem cells can migrate to damaged tissues or organs, and in the treatment of kidney diseases, they can differentiate directly into intrinsic renal cells or secrete a series of bioactive molecules through paracrine mechanisms, regulate the microenvironment, inhibit inflammatory reactions, and alleviate kidney tissue damage [107,108]. In recent years, many scholars have explored the effectiveness and mechanism of stem cell therapy for DKD.

### 5.1. Mesenchymal Stem Cell Therapy

Common stem cell therapies mainly utilize MSCs, which not only have the ability to migrate to damaged tissue sites but also have strong immunosuppressive effects [105]. Therefore, the use of mesenchymal cells is expected to become an effective method for treating DKD. According to their isolation sources, MSCs can be derived from adipose tissue, bone marrow, placenta, or the umbilical cord [106].

#### 5.1.1. Bone Marrow-Derived Mesenchymal Stem Cells

Bone marrow mesenchymal stem cells (BMSCs) can be obtained from bone marrow by isolating monocytes. In recent years, many scholars have explored the efficacy and mechanism of BMSCs in treating DKD. BMSCs can also exert renoprotective effects by regulating the levels of blood glucose. Lv et al. reported that BMSCs could inhibit glucose uptake mediated by GLUT1 and lower blood glucose levels, thereby suppressing oxidative stress, reducing urinary albumin excretion, and improving glomerulosclerosis [109].

Pan et al. transplanted BMSCs into diabetic tree shrews via intravenous infusion, which increased their insulin level and decreased their postprandial blood glucose, blood creatinine and urea nitrogen levels [110]. In addition, BMSCs alleviate DKD by regulating immune cells. BMSCs can significantly downregulate the expression of transcription factors necessary for the development of CD103+ DCs, inhibit DC maturation, reduce the expression of inflammatory factors and chemokines, inhibit CD8+ T-cell proliferation, and confer renal protection [111]. BMSCs promote macrophage differentiation into the M2 phenotype and alleviate the inflammatory response in DKD model mice by activating the transcription factor EB [112]. In addition, Lv et al. reported that the implantation of BMSCs could effectively reduce blood glucose and improve renal function in DKD mice and could also inhibit the TGF-β/Smad signaling pathway through the secretion of bone morphogenetic protein 7, improving glomerular fibrosis [113].

#### 5.1.2. Umbilical Cord Mesenchymal Stem Cells

MSCs derived from the umbilical cord have lower immunogenicity, greater proliferation potential and differentiation ability, a faster self-renewal ability, and broader application prospects [114]. In STZ-induced diabetic mice, Li et al. reported that repeated injection of umbilical cord MSCs could inhibit TGF-β1-triggered myofibroblast differentiation through paracrine action and increase the levels of MMP2 and MMP9, thereby reducing the deposition of fibronectin and collagen I and alleviating proteinuria, glomerular injury, and fibrosis in DKD mouse models [115]. In addition to inhibiting fibrosis during the process of DKD, scholars have reported that MSCs in DKD could reduce cell death and delay disease progression through antiapoptotic effects [116]. Moreover, MSCs prevent the progression of DKD by inducing Arg1 expression in macrophages, which subsequently reduces mitochondrial dysfunction in tubular epithelial cells [117], to improve blood glucose control and anti-inflammatory activity [118].

#### 5.1.3. Placenta-Derived Mesenchymal Stem Cells

Placenta-derived mesenchymal stem cells (PMSCs) are favored because of their accessibility and lack of associated ethical issues [119]. Wang et al. reported that PMSCs could be recruited into immune organs to promote Th17/Treg balance in the kidneys and blood of DKD rats by acting on the PD-1/PD-L1 pathway, which reduced the levels of proinflammatory cytokines (IL-17A and IL-1β) and improved renal function and pathological damage in DKD rats [120]. In addition, Han et al. reported that PMSCs could also regulate molecular pathways to exert renoprotective effects. Injecting PMSCs into DKD rats activated the expression of the SIRT1-PGC-1α-TFAM pathway to alleviate podocyte damage and mitochondrial autophagy inhibition [121].

#### 5.1.4. Adipose-Derived Mesenchymal Stem Cells

Because of their abundance in vivo, adipose-derived mesenchymal stem cells (ADMSCs) have become an attractive cell source [122,123]. ADMSC transplantation may alleviate cell apoptosis and improve renal histology to alleviate DKD damage by activating klotho and inhibiting the Wnt/β-catenin pathway [124]. Yang et al. investigated the effect of ADMSCs combined with empagliflozin (EMPA) on DKD, and the results showed that ADMSC-EMPA combined therapy protected renal function and ultrastructural integrity in DKD mice better than monotherapy [125]. However, the retention and survival rates of ADMSCs are low in the kidneys. Therefore, scholars constructed ADMSC sheets to extend the survival time of ADMSCs in the kidneys, which allowed these cells to play a protective role in maintaining the renal tubular structure [126].

#### 5.1.5. Mesenchymal Stem Cell-Derived Exosomes

Exosomes are extracellular vesicles with a diameter of 30 to 150 nanometers secreted by MSCs [127]. In kidney disease, in addition to their ability to function as stem cells themselves, MSC-derived exosomes can carry complex molecular cargo such as proteins, lipids, and nucleic acids (DNA, miRNA, and circRNA), to regulate cell apoptosis, proliferation, and immune response [108,128]. At present, extracellular vesicles from different sources of MSCs have been studied in DKD. Ebrahim et al. reported that BMSC-derived exosomes improved the autophagy of tubular cells in DKD mice by inhibiting the expression of rapamycin targets, reducing fibrosis, and improving kidney function [129]. The intravenous injection of extracellular vesicles derived from human umbilical cord MSCs could reduce blood glucose levels and improve insulin resistance in diabetic rats, indicating their potential application value in DKD [130]. Extracellular vesicles derived from ADMSCs have the potential to become intervention targets for DKD treatment by alleviating podocyte apoptosis, reducing the level of blood glucose in DKD mice, and inhibiting mesangial proliferation and renal fibrosis [131,132].

### 5.2. Other Types of Stem Cell Therapies

Induced pluripotent stem cells (iPSCs) are pluripotent cells formed by reprogramming terminally differentiated somatic cells with specific transcription factors, similar to human embryonic stem cells. Given the high differentiation ability of iPSCs, they can be induced to differentiate into renal tubular cells and podocytes [133]. Raikwar et al. generated endodermal cells from human iPSCs by treating them with activin A and induced them to differentiate into three-dimensional islet cell clusters through a series of growth factors. IPSCs reportedly play a vital role in improving the blood glucose level of diabetic mice [134]. Embryonic stem cells (ESCs) are a type of multipotent cell derived from the cell population within the blastocyst that can be induced into renal cells through a series of determined growth factors or inducers [135,136], indicating the potential for transplantation and regenerative therapy of damaged kidneys [108]. Currently, research on the treatment of DKD with iPSCs and ESCs remains in the early experimental stage. In addition, pancreatic Ngn3 stem cells can differentiate into mature beta cells within the pancreas, which can secrete insulin and maintain blood glucose stability in the body [137]. Intensive insulin therapy can reduce the occurrence of microalbuminuria, pancreatic stem cells may become the cell source for pancreatic islet formation [138,139], providing a new approach for the treatment of DM.

Stem cells have a wide range of sources, and autologous stem cells do not have immune rejection reactions, providing prospects for clinical applications. However, further research is needed to evaluate their therapeutic effects. The transplantation of stem cells will provide more options for the treatment of DKD.

## 6. Gut Microbiota-Targeted Therapy

The plethora of different types of bacteria in the intestine are collectively referred to as the gut microbiome. The disturbance of the intestinal flora plays an important role in the pathogenesis of DKD, which may be attributed to several factors [140]. For example, uremia in patients with CKD affects the composition and metabolism of the intestinal flora. Intestinal ecological imbalance may cause damage to the epithelial barrier, ultimately leading to increased exposure of the host to endotoxins [141]. Moreover, the large amount of proinflammatory and nephrotoxic substances produced by gut microbiota can trigger local inflammation in the kidneys, leading to a decrease in the eGFR [142]. Uremic toxins, such as p-cresyl sulfate, trimethylamine-N-oxide, and indoxyl sulfate, originate from microbial metabolism, and their accumulation exacerbates damage to the kidneys and cardiovascular system [143]. Intervening in the human gut microbiome is expected to become an effective treatment for alleviating DKD. Currently, many scholars are focusing on the role of regulating the gut microbiome to treat DKD, mainly with dietary strategies, probiotic and prebiotic treatment, and fecal microbiota transplantation (FMT) methods.

Diet is the main exogenous factor affecting the composition of the gut microbiome. Short-chain fatty acids (SCFAs), which are considered to play a key role in microbial host crosstalk, are the main metabolites produced by bacterial fermentation of dietary fiber. Research has shown that SCFAs can alleviate the inflammatory response in renal ischemia–reperfusion injury [144]. Li et al. reported that a high-fiber diet could improve the degree and probability of DKD development in a mouse model. Dietary fiber can prevent DKD by regulating the intestinal flora, enriching bacteria that produce SCFAs and increasing the production of SCFAs [145].

Probiotics and prebiotics are commonly used to regulate the gut microbiome. Probiotics are living microorganisms that synthesize multiple vitamins to improve the balance of host gut microbiota. Prebiotics, regulating the composition of gut microbiota and benefiting the human health, are ingredients of fermented food [146]. Many scholars have reported the positive effects of probiotic or prebiotic supplementation in CKD patients, including reduced uremic toxins and blood glucose levels, a restructured gut microbiome, and reductions in oxidative stress and inflammation [146,147]. In DKD patients, Dai et al. reported that probiotics could delay the progression of renal dysfunction, improve glucose and lipid metabolism, and reduce symptoms and oxidative stress [148].

Intestinal FMT is a process by which the gut microbiome of a healthy donor is transferred into a recipient to introduce or restore a stable gut microbiome. Previous studies have shown that FMT can prevent diabetic patients from gaining weight, reduce proteinuria and local intestinal inflammation, and improve insulin resistance [149]. Shang et al. reported that the level of uremic toxins in DKD mice significantly decreased and that DKD lesions gradually improved after the transplantation of feces from healthy mice [150]. In a mouse model of DKD, Bastos et al. reported that FMT could prevent weight gain, reduce albuminuria and local intestinal inflammation, and improve insulin resistance [149]. Because the mechanisms underlying the effects of FMT in humans are complex, the clinical applicability of FMT needs to be verified in clinical trials.

In general, the understanding of the relationship between the gut microbiome and DKD is in its infancy. It is still unclear which gut microbiota play a beneficial role and which play a harmful role in DKD. Further research is needed on the dosage, treatment duration, and long-term effectiveness of various bacterial colonies. Before gut microbiota-targeted therapy can be systematically used to treat or prevent DKD, additional research is needed [151].

## 7. Lifestyle Intervention

Except for the therapy methods discussed above, lifestyle management has also gradually gained attention in the treatment of DKD, including smoking cessation, exercise, weight loss and so on. Obesity can increase insulin resistance, activate inflammation and increase oxidative stress [152].Bolignano et al. found that weight loss could significantly reduce albuminuria and proteinuria in diabetic patients undergoing weight loss surgery [153].Therefore, weight loss is an important method to treat DKD. Physical activity is a basic component of lifestyle management in diabetic care. Research found that regular moderate and high-intensity physical activity is associated with a reduction in incidence rate and progression of DKD, as well as cardiovascular events and mortality. A comparative study in CKD patients (including DKD patients) suggested that exercise could reduce the decline in eGFR by approximately 0.5% annually for every 60-min increase in weekly activity duration [154].Smoking can lead to the progression and deterioration of DKD. Fedoroff et al. found that smoking is a risk factor for the progress of DKD, and the risk is increased with increased smoking dose [155]. Quitting smoking has been recommended in KDIGO for DKD patients [156]. Lifestyle intervention is also an important treatment method for DKD patients.

## 8. Conclusions

Several challenges exist in the molecular diagnosis and treatment of DKD. Firstly, the description of endpoints in DKD mainly include a decline in eGFR, the need for dialysis or transplant, and mortality. It is of great significance to explore more rational evaluation indicators of endpoints for DKD, such as proteinuria, to assist in evaluating the effectiveness of clinical interventions. Secondly, different phenotypes including proteinuric and non-proteinuric manifestations exist in DKD, and the specific diagnosis and treatment of the two different phenotypes requires further exploration. Thirdly, novel biomarkers that can not only predict the progression of DKD but also accurately reflect the response to current treatments are needed. However, novel biomarkers applied in clinical practice are still scarce and require further research.

**Figure 1:**
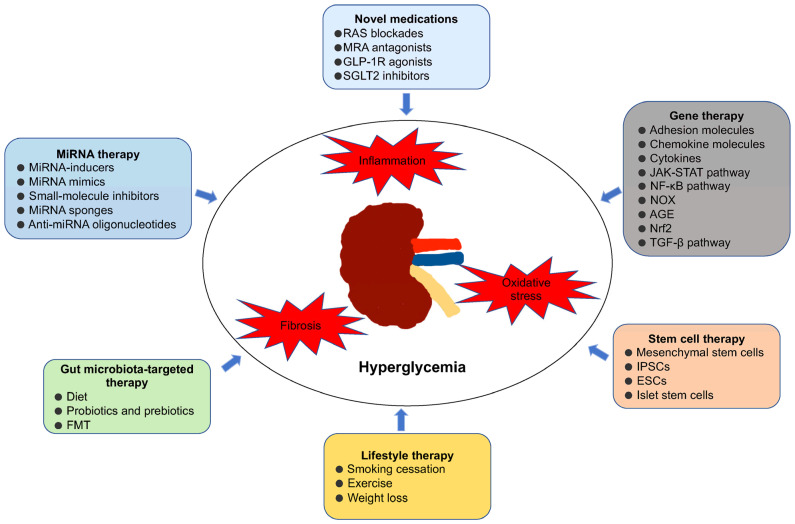
Current and potential targets for therapeutic interventions of DKD. Abbreviations: RAS: renin–angiotensin–aldosterone system; MRAs: mineralocorticoid system; SGLT2: sodium–glucose cotransporter 2; GLP-1R: glucagon-like peptide 1 receptor; JAK/STAT: Janus kinase/signal transducer and activator of transcription; NOX: nicotinamide adenine dinucleotide phosphate oxidases; AGE: advanced glycation end product; Nrf2: nuclear factor erythroid 2-related factor 2; IPSC: induced pluripotent stem cells; ESC: embryonic stem cell; FMT: fecal microbiota transplantation.

Currently, although several novel drugs, such as RAS blockers, SGLT2 inhibitors, and MRAs, have shown clinical benefits for DKD, the current treatment options still have a limited ability to prevent the progression of kidney disease and reduce the risk of complications and death in DKD patients. Therefore, it is highly important to explore new treatment methods and optimize treatment strategies for controlling the progression of DKD. This review introduces new treatment strategies, including gene therapy, stem cell therapy, miRNA therapy, gut microbiota-targeted therapy and lifestyle intervention, which have the potential to usher in a new era of DKD management that improves angiopathy, kidney function, and survival rates (Figure 1). However, more multicenter, large-scale prospective clinical studies are urgently needed to verify the prognostic impact of these novel therapeutics on DKD patients. Hopefully, an increasing number of drugs will enter clinical trials to provide new ideas for the treatment and intervention of DKD and to inhibit the development of DKD into ESRD.

## Figures and Tables

**Table 1:** Summary of selected clinical trials evaluating molecular therapies with anti-inflammatories and antioxidants in DKD.

Drug	Proposed Effect	Kidney Outcome	Reference
ASP8232	Inhibit the activity of VAP-1, improve oxidative stress damage and cellular toxicity	Reduced urinary ACR	[32]
Emapticap pegol	Inhibit the pro-inflammatory chemokine C-C motif-ligand 2	Improved the ACR and HbA1c	[35]
Baricitinib	Inhibit the inflammation signal JAK1/JAK2	Decreased albuminuria	[46]
Gevokizumab	Anti-IL-1β antibody	Improved glycemia, decreased inflammation in diabetic patients	[41]
Bardoxolone methyl	An activator of Nrf2	Improved renal function, but the study was terminated because of increased early heart failure events	[61]
Pirfenidone	Inhibit the activity of TGF-β1,2,3	Increased eGFR	[65]
Pentoxifylline	Downregulate NF-κB signaling	Reduced proteinuria	[51]
GKT137831	Inhibitor of NOX 1/4	Failed to reduce albuminuria	NCT02010242
Canakinumab	Inhibitor of IL-1β	Neither clinical benefit nor substantial harm	[42]
Green tea	Interference with AGE receptor	No status of the study	NCT03622762

ACR: albumin-to-creatinine ratio, HbA1c: glycosylated hemoglobin type A1C, VAP-1: vascular adhesion protein-1, Nrf2: nuclear factor erythroid 2-related factor 2, JAK: Janus kinase, NOX: Nicotinamide adenine dinucleotide phosphate oxidase, eGFR: estimated glomerular filtration rate, AGE: advanced glycation end product.

## Data Availability

Not applicable.
